# Growing Menace of Antibacterial Resistance in Clinical Isolates of *Pseudomonas aeruginosa* in Nepal: An Insight of Beta-Lactamase Production

**DOI:** 10.1155/2016/6437208

**Published:** 2016-08-23

**Authors:** Shamshul Ansari, Rabindra Dhital, Sony Shrestha, Sangita Thapa, Ram Puri, Niraj Chaudhary, Suresh Khatiwada, Rajendra Gautam

**Affiliations:** ^1^Department of Microbiology, Chitwan Medical College, Bharatpur, Chitwan, Nepal; ^2^Department of Laboratory Medicine, Chitwan Medical College, Bharatpur, Chitwan, Nepal

## Abstract

*Introduction. Pseudomonas aeruginosa* is the most frequently isolated organism as it acts as the opportunistic pathogen and can cause infections in immunosuppressed patients. The production of different types of beta-lactamases renders this organism resistant to many commonly used antimicrobials. Therefore, the aim of this study was to document the antibiotic resistance rate in* Pseudomonas aeruginosa* isolated from different clinical specimens.* Methods. Pseudomonas aeruginosa* recovered was identified by standard microbiological methods. Antibiotic susceptibility testing was performed by modified Kirby-Bauer disc diffusion method following Clinical and Laboratory Standard Institute (CLSI) guidelines and all the suspected isolates were tested for the production of ESBLs, MBLs, and AmpC.* Results.* Out of total (178) isolates, 83.1% were recovered from the inpatient department (IPD). Majority of the isolates mediated resistance towards the beta-lactam antibiotics, while nearly half of the isolates were resistant to ciprofloxacin. Most of the aminoglycosides used showed resistance rate up to 75% but amikacin proved to be better option. No resistance to polymyxin was observed. ESBLs, MBLs, and AmpC mediated resistance was seen in 33.1%, 30.9%, and 15.7% isolates, respectively.* Conclusions*. Antibiotic resistance rate and beta-lactamase mediated resistance were high. Thus, regular surveillance of drug resistance is of utmost importance.

## 1. Introduction

Among the nonfermenters,* Pseudomonas aeruginosa* (*P. aeruginosa*) is the most frequently isolated organism from clinical cases.* P. aeruginosa* is the opportunistic pathogen that frequently caused the infections of immune-compromised patients and also is involved in the outbreaks of hospital acquired infections with high mortality rate [1, 2]. This organism is innately resistant to many classes of antibiotics. Therefore, an infection caused by this organism is difficult to treat because of the high degree of antimicrobial resistance [3]. The tendency of this organism to develop the antibiotic resistance is the current global concern regarding antimicrobial therapy [4].

There are varieties of antibacterial resistance mechanisms which are involved in the resistance development in* P. aeruginosa*; among them, overexpression of efflux pumps [5] and acquisition of beta-lactamases, that is, extended spectrum beta-lactamases and metallo-beta-lactamases [4], are the predominant mechanisms. Beta-lactamases are the enzymes that are encoded by the chromosomal and plasmid genes of many bacteria [6]. Beta-lactamases are produced as a form of metabolic by-products that have the ability to hydrolyze and destroy the beta-lactam antibiotics [7]. Resistance mediated by extended spectrum beta-lactamases (ESBLs), metallo-beta-lactamases (MBLs), and AmpC beta-lactamase (AmpC) enzymes to broad-spectrum beta-lactam antibiotics is the global threat for the antimicrobial therapy [8]. Therefore, the increasing trend of the antibiotic resistance due to beta-lactamase enzymes to commonly used antibacterial agents for the treatment of infections caused by* P. aeruginosa *and its current impact on antibacterial therapy failure encouraged us to find out the beta-lactamases mediated resistance rate and current antibiotic options for effective treatment of this organism. To the best of our knowledge, this is one of the few studies with exclusive focus on investigating the antimicrobial susceptibility pattern on ESBL, MBL, and AmpC producing* P. aeruginosa*.

## 2. Methods

### 2.1. Study Type and Area

A cross-sectional study was carried out from February to July 2015 at Chitwan Medical College, Bharatpur, Chitwan, Nepal. This 600-bed teaching hospital is a tertiary care center in the city of Bharatpur, Chitwan district of Nepal.

### 2.2. Bacterial Isolates

A total of 178 nonrepetitive and nonredundant clinical isolates of* P. aeruginosa* (pus 32, sputum 68, urine 42, blood 20, endotracheal tube 8, swab 4, bronchoalveolar lavage 2, and pleural fluid 2) from inpatient department (IPD) and from outpatient department (OPD) were included in this study. All the bacterial isolates recovered were identified by standard microbiological methods recommended by American Society for Microbiology (ASM) [9].

### 2.3. Antimicrobial Susceptibility Testing

Antimicrobial susceptibility testing was performed by the modified Kirby-Bauer disk diffusion method on standard Muller-Hinton agar medium using commercially prepared antibiotic disks according to the Clinical and Laboratory Standard Institute (CLSI) guidelines and interpretation of antibiotic susceptibility result was made according to the zone-interpretative chart provided by CLSI [10]. Antibiotics used were ceftazidime (30 *μ*g), ceftriaxone (30 *μ*g), cefotaxime (30 *μ*g), cefixime (5 *μ*g), imipenem (10 *μ*g), meropenem (10 *μ*g), carbenicillin (100 *μ*g), piperacillin (100 *μ*g), piperacillin-tazobactam (100/10 *μ*g), ciprofloxacin (5 *μ*g), gentamicin (10 *μ*g), amikacin (30 *μ*g), tobramycin (10 *μ*g), and polymyxin B (10 *μ*g).* Escherichia coli* (ATCC 25922) and* Pseudomonas aeruginosa (*ATCC 27853) were used as the reference strains throughout the study.

### 2.4. Phenotypic Detection of Extended Spectrum Beta-Lactamases (ESBLs)

Screening of ESBLs production was carried out according to the NCCLS guidelines [11]. Briefly, the isolates were subjected to susceptibility to third-generation cephalosporins using ceftazidime (30 *μ*g) and cefotaxime (30 *μ*g) disks. The isolates that showed the zone of inhibition of diameter of ≤22 mm for ceftazidime and/or ≤27 mm for cefotaxime were screened as ESBLs producers. All isolates that found positive screening for ESBLs production were subjected to confirmation of ESBLs production by combination disk method on standard Muller-Hinton agar medium using standardized inoculum of the isolate. After standardization, the broth cultures of strains with 0.5 McFarland standards were inoculated on standard Muller-Hinton agar media to form a lawn culture. Commercial antibiotic disks containing ceftazidime (30 *μ*g) and cefotaxime (30 *μ*g) with and without clavulanic acid were used in this method. An increase in diameter of ≥5 mm for ceftazidime with clavulanic acid or cefotaxime with clavulanic acid compared with ceftazidime or cefotaxime disk alone, respectively, was considered positive for ESBLs as described by Carter et al. [12].

### 2.5. Phenotypic Detection of Metallo-Beta-Lactamases (MBLs)

Screening of MBLs production was carried out by using imipenem, meropenem, or third-generation cephalosporin (ceftazidime) disks. The isolates that showed reduced susceptibility to imipenem, meropenem, or ceftazidime in Kirby-Bauer disk diffusion method were presumptively taken as MBLs producers and were confirmed by disk potentiation method. Briefly, inocula of test strains were prepared compared with 0.5 McFarland standards and inoculated on standard Muller-Hinton agar media to form a lawn culture. Two imipenem disks were placed on the plate at standard distance and 10 *μ*L of ethylene diamine tetra-acetic acid (EDTA) solutions was added to one imipenem disk to achieve a desired concentration of 750 *μ*g. The diameters of zone of inhibition of imipenem and imipenem with EDTA were compared after incubation of 16–18 hours at 35°C. An increase of ≥7 mm in diameter of zone of inhibition for imipenem with EDTA disk compared with imipenem disk alone was identified as MBLs production as described by Yong et al. [13].

### 2.6. Phenotypic Detection of AmpC Beta-Lactamase (AmpC)

All strains with reduced susceptibility to cefoxitin in modified Kirby-Bauer disk diffusion method were confirmed for AmpC beta-lactamase production using the method described by Singhal et al. [8]. In this method, a cefoxitin susceptible* Escherichia coli* indicator strain (ATCC 25922) was inoculated on standard Muller-Hinton agar medium to form a lawn culture and a cefoxitin disk was placed. A blank disk of 6 mm diameter moistened with normal saline was inoculated with few colonies of test strain and placed next to the cefoxitin disk. The plate was incubated at 37°C overnight. After overnight incubation, indentation in the cefoxitin inhibition zone adjacent to the disk containing test strain was an indication of the AmpC beta-lactamase production [8].

### 2.7. Phenotypic Characterization of Multitype Enzymes (Beta-Lactamases) Production

The isolates showing the positive result for at least two beta-lactamases methods described above (ESBLs, MBLs, and AmpC) were characterized as multitype enzyme producing isolates.

### 2.8. Ethical Consideration

The bacterial strains used in this study were isolated from the routine clinical specimens and verbal consent was obtained from the patients. This study was approved by the Institutional Review Committee of Chitwan Medical College (IRC-CMC), Bharatpur, Chitwan, Nepal.

## 3. Results

### 3.1. Patients' Characteristics

During the study period, 178* P. aeruginosa* isolates were recovered from a variety of specimens collected at the bacteriology department of Chitwan Medical College Teaching Hospital (CMCTH). Out of 178 isolates, 104 (58.4%) were isolated from male patients, while 74 (41.6%) were recovered from female patients. Majority of the cases (83.1%) were from inpatient department (IPD), while 16.9% of cases were from outpatient department (OPD) of the hospital (Table 1).

### 3.2. Isolates and Specimen Types

In this study, we collected a variety of specimens such as pus (wound infections), sputum, urine, blood, endotracheal tube sample, swabs (other than wound infections), bronchoalveolar lavage, and pleural fluid. The majority of the isolates were from sputum (68) followed by urine (42) and pus (32), while a minor number of isolates were contributed by the bronchoalveolar lavage (2) and pleural fluid (2) samples (Table 1).

### 3.3. Antibacterial Susceptibility Profile

Among tested antibiotics, the beta-lactams were found to be less effective in* P. aeruginosa*. The highest rate of resistance (78.6%) was mediated towards piperacillin, whereas more than half of the isolates were resistant to other beta-lactam antibiotics. Nearly half of the isolates were resistant to fluoroquinolone (ciprofloxacin). Aminoglycosides (gentamicin and tobramycin) also showed resistance rate up to 75%, whereas amikacin showed promising efficacy, showing resistance rate below 30% in isolates from all specimen types. Polymyxin B was found to be the best regimen for the treatment of infections caused by* P. aeruginosa*, as polymyxin B resistance was not documented in this study (Table 2).

### 3.4. Enzyme (Beta-Lactamases) Producing Isolates

Enzyme mediated drug resistance was frequently observed in this study. ESBLs mediated resistance was observed in 59 (33.1%) isolates. The majority of ESBLs mediated resistant isolates were recovered from bronchoalveolar lavage sample (100%) followed by swab sample (50%) and pus sample (40.6%), while there was no ESBLs mediated resistance seen in isolates recovered from pleural fluid (Table 3).

Out of total isolates recovered, MBLs mediated resistance was observed in 55 (30.9%) isolates. The majority of MBLs positive isolates were recovered from endotracheal tube sample (75%) followed by urine sample (33.3%), while there was no MBL mediated resistance seen in isolates from bronchoalveolar lavage and pleural fluid (Table 3).

AmpC mediated antibiotic resistance was reported in 15.7% of isolates, with the highest proportions of isolates being from endotracheal tube sample (100%) followed by swab sample (50%), while there was no AmpC positive isolate from bronchoalveolar lavage and pleural fluid samples (Table 3).

In our study, no enzyme mediated drug resistance was found in isolates from pleural fluid specimens and this may be because of small sample size (Table 3).

### 3.5. Multitype Enzymes Production

In current study, we also identified few isolates harboring multitypes of enzymes mediating antibacterial resistance. ESBLs together with MBLs and ESBLs together with AmpC combinations mediating resistance were identified from 6 (3.4%) isolates each, while the combination of MBLs together with AmpC mediated resistance from 14 (7.9%), whereas ESBLs together with MBLs and AmpC mediated resistance from 2 (1.1%) isolates (Table 4).

## 4. Discussions

The development of resistance to multiple antibiotics in microbes is in increasing trend which is the leading cause of treatment failure and is a serious problem of global magnitude. The organisms like* P. aeruginosa*, methicillin resistant* Staphylococcus aureus* (MRSA), vancomycin resistant* Enterococcus* (VRE), glycopeptide intermediate* Staphylococcus aureus* (GISA), glycopeptide resistant* Staphylococcus aureus* (GRSA),* Acinetobacter baumannii*, and* Stenotrophomonas maltophilia* belong to multidrug resistance organisms and therefore need special attention as they are commonly isolated from health care associated infections [14].

The immunosuppressed and chronic lung disease patients often develop hospital-acquired pneumonia.* P. aeruginosa* is commonly isolated from the respiratory tracts of cystic fibrosis patients and is often a cause of severe decline in these patients. Chronic lung colonization and infections by this organism also occur in patients with diseases affecting the airways of the lungs such as bronchiectasis and chronic obstructive pulmonary disease [15]. We also recovered the majority of the isolates from lower respiratory tract specimen (sputum). The highest recovery rate from sputum in our study also correlates with the study conducted by Khan et al. (Nepal) in 2014 [16]. In the present study, among total enrolled isolates, as high as 83.1% of the isolates were recovered from the IPD patients. Similarly, high rate of* P. aeruginosa* was also reported from IPD patients by several authors such as 81.6% by Basak et al. (India) [4] and 61% by Algun et al. (Turkey) [17].

*P. aeruginosa* is one of the most important microorganisms in clinical settings which cause problems as a result of its high resistance to antimicrobial agents and therefore it is a dangerous and dreaded bug.* P. aeruginosa*, with the profound use of various antibiotics, has changed itself into a stubborn organism, resistant to almost all the antibiotics. Beta-lactams are the broad group of antibiotics that contain a beta-lactam ring in their molecular structures [18]. Bacteria often develop resistance to beta-lactam antibiotics by producing beta-lactamase, an enzyme that can break the beta-lactam ring. Previously, these antibiotics were mainly effective against Gram-positive bacteria, but with the recent development of broadening the spectrum, these antibiotics show activity against various Gram-negative organisms including* P. aeruginosa*. As a consequence of low cost and indiscriminate use, these antibiotics have decreased their usefulness in Nepal. In this study also we found that the beta-lactam antibiotics were least effective. All the beta-lactam antibiotics tested in this study showed resistance rate up to 100%, much higher than the study conducted in Nepal by Mishra et al. in 2012 [19], Sherchan et al. in 2012 [20], and Khan et al. in 2014 [16]. Similarly, the findings of our study corroborate the observations of other authors such as Rafiee et al. (Iran) [21], Basak et al. [4], and Behera et al. from India [22].

Fluoroquinolones have been used extensively for the treatment of both minor and more serious infections. As a consequence, their role today is increasingly limited due to increasing resistance which poses a great therapeutic challenge in Nepal. We identified resistance rate to ciprofloxacin in as high as 85% of the isolates, higher than previous studies conducted in Nepal by Shankar et al. in 2005 [23], Bhatt and Lakhey in 2006 [24], Mishra et al. in 2012 [19], Baral et al. in 2012 [25], Sherchan et al. in 2012 [20], Chander and Raza in 2013 [26], and Khan et al. in 2014 [16]. Similar trend of resistance was also reported to aminoglycosides (gentamicin, amikacin, and tobramycin), being resistant up to 75% to gentamicin in our study, which was comparably higher than other studies from Nepal (33.3% in 2005 [23], 60% in 2006 [24], 34% in 2012 [19], and 46.9% in 2014 [16]). Amikacin was found somewhat as an effective regimen showing resistance rate up to 28%, even being a higher rate of resistance when compared to other observations such as 0% by Shankar et al. in 2005 [23], 15.78% by Sherchan et al. in 2012 [20], 22% by Mishra et al. in 2012 [19], and 17.25% by Chander and Raza in 2013 [26].

In this study, one striking feature was that no isolates showed resistance to cytoplasmic membrane damaging agents (polymyxin), confirming that polymyxin B should be used as the empiric therapy for serious pseudomonal infections. Similarly, Agrawal et al. in 2008 [27] and Khanal et al. in 2013 [28] also found polymyxin B as the most effective regimen.

In their natural habitats, most of* P. aeruginosa* do not produce slime layer of alginate polysaccharide (nonmucoid) and this leads to the reduction of the biofilm production rendering vulnerable to antibiotics. However, the production of biofilm leads the organisms to be resistant to many antibiotics [29]. Several mechanisms that contribute to resistance to beta-lactam antibiotics in* P. aeruginosa* include genetic mutation leading to stable overexpression of chromosome-mediated AmpC cephalosporinases, acquisition of transferable beta-lactamase genes, overproduction of efflux systems, and reduced permeability [30]. Ambler class ESBLs and MBLs are reported as rapidly growing enzymes in clinical isolates of* P. aeruginosa* which can lead to multidrug resistance [31]. More than 800 beta-lactamases have been identified in Gram-negative bacteria and at least 120 types of these were detected in* P. aeruginosa*, among which ESBLs, MBLs, and AmpC are clinically significant [32]. The reduced susceptibility of Gram-negative isolates to the later generation cephalosporins could be attributable to ESBL or AmpC *β*-lactamase production or some other relevant underlying mechanisms. In the current study, we observed high prevalence of the ESBLs mediated resistance in 33.1% of isolates and it was observed in 100% of isolates from bronchoalveolar lavage. Our rate of ESBLs mediated resistance was found to be much higher in comparison to one other study carried out in Nepal (2.4% by Shrestha et al. in 2011 [33]) but was similar to Khanal et al. (2013) [28].

Carbapenems are mostly used as the last resort for treatment of multidrug resistance bacterial infections. However, MBLs mediated acquired resistance to this life saving antimicrobial has been increasingly reported in many types of Gram-negative bacteria including* P. aeruginosa* in the last 15 years [34]. In the current study, out of total 178* P. aeruginosa* isolates, we identified MBLs mediated resistance in 30.9% of isolates, much higher than previous studies carried out by several authors from Nepal (2.4% by Shrestha et al. in 2011 [33], 3.3% by Mishra et al. in 2012 [19], and 18.2% by Khanal et al. in 2013 [28]). Lower rate of MBLs mediated resistance was also reported by Padiyath et al. (16%) [35] and Chaudhary and Payasi (16.9%) [36] from India.

The current CLSI guidelines do not describe a method for detection of AmpC beta-lactamase. AmpC disc test was originally introduced to detect plasmid-mediated AmpC beta-lactamases [37]. However, Black et al. reported the detection of chromosomally mediated inducible AmpC beta-lactamases in a number of bacteria including* P. aeruginosa* by the AmpC disc test method [7]. AmpC mediated resistance was observed in 15.7% of isolates in our study which was comparable to 17.6% observed by Khanal et al. in 2013 [28].

The combination of multitypes of beta-lactamase production in* P. aeruginosa *can cause major treatment failure and can pose significant clinical challenges if it remains undetected [38]. Therefore, detection of combination of multiple beta-lactamases in highly resistant bacteria could be useful for the selection of suitable antibiotic therapy and avoiding treatment failure as well as reducing mortality rates in hospitalized patients. In this study, several combinations of multiple types of beta-lactamases were observed. We identified coproduction of ESBLs together with MBLs in 3.4%. Similar finding (4%) of ESBLs together with MBLs was also documented by Padiyath et al. from India [35] but higher rate (14.4%) of coproduction of ESBLs together with MBLs was documented by Chaudhary and Payasi from India [36]. Coproduction of ESBLs and AmpC in this study (3.4%) was similar to the finding of Rafiee et al. (3.9%) [21] but was much lower than that (26%) reported by Padiyath et al. from India [35]. We also found coproducers of MBLs together with AmpC as much as 7.9%. Production of AmpC together with ESBLs and MBLs was observed in 1.1% of isolates in the present study.

In the present study, we found that almost all isolates were resistant to antibiotics. Most of the isolates were producers of beta-lactamases, which renders isolates resistant to cell wall inhibiting agents. However, this study could not omit certain limitations as we were unable to conduct the molecular characterization for various beta-lactamases production and we also failed in looking for other resistance mechanisms such as efflux pump system, as this also can mediate resistance in* P. aeruginosa*.

## 5. Conclusions

This study reports the high prevalence of drug resistance and comparable rates of ESBLs, MBLs, and AmpC mediated resistance in* P. aeruginosa* in our setting. Therefore, there is a need for longitudinal and nationwide surveillance for drug resistance in clinical isolates of* P. aeruginosa*. Strict hospital infection control policies for the prudent use of antimicrobials are to be adapted to minimize the antimicrobial resistance.

## Figures and Tables

**Table 1:** Gender- and department-wise distribution of positive specimens.

Specimens type (number of isolates)	Male	Female	IPD	OPD
Pus (wound infection) (32)	16	16	26	6
Sputum (68)	42	26	64	4
Urine (42)	34	8	28	14
Blood (20)	6	14	14	6
Endotracheal tube sample (8)	6	2	8	0
Swab (other than wound infection) (4)	0	4	4	0
Bronchoalveolar lavage (2)	0	2	2	0
Pleural fluid (2)	0	2	2	0

*Total (178)*	*104 (58.4*%)	*74 (41.6*%)	*148 (83.1*%)	*30 (16.9*%)

**Table 2:** Antibacterial resistance rates of *Pseudomonas aeruginosa* isolates.

Antibiotics	Pus (32) Number (%)	Sputum (68) Number (%)	Urine (42) Number (%)	Blood (20) Number (%)	ET-tube (8) Number (%)	Swab (4) Number (%)	BAL (2) Number (%)	Pleural fluid (2) Number (%)	Total (178) Number (%)
CAZ	24 (75)	56 (82.3)	30 (71.4)	9 (45.0)	6 (75.0)	3 (75.0)	2 (100)	0	130 (73.0)
CTR	12 (37.5)	30 (44.1)	26 (61.9)	12 (60.0)	6 (75.0)	4 (100)	2 (100)	0	92 (51.7)
CTX	24 (75)	26 (38.2)	34 (80.9)	8 (40.0)	6 (75.0)	4 (100)	0	0	102 (57.3)
CFM	16 (50)	59 (86.8)	32 (76.2)	16 (80.0)	8 (100)	3 (75.0)	0	2 (100)	134 (75.3)
IPM	12 (37.5)	28 (41.2)	18 (42.8)	4 (20.0)	8 (100)	1 (25.0)	0	0	71 (40.0)
MRP	12 (37.5)	28 (41.2)	16 (38.1)	6 (30.0)	8 (100)	1 (25.0)	0	0	71 (40.0)
CB	12 (37.5)	18 (26.5)	26 (61.9)	4 (20.0)	4 (50.0)	2 (50.0)	2 (100)	0	68 (38.2)
PI	24 (75)	55 (80.9)	32 (76.2)	13 (65.0)	8 (100)	4 (100)	2 (100)	2 (100)	140 (78.6)
PIT	16 (50)	21 (30.9)	16 (38.1)	6 (30.0)	5 (62.5)	2 (50.0)	1 (50.0)	2 (100)	69 (38.8)
CIP	8 (25)	30 (44.1)	36 (85.7)	11 (55.0)	6 (75.0)	0	0	0	91 (51.1)
GEN	12 (37.5)	8 (11.8)	14 (33.3)	8 (40.0)	6 (75.0)	2 (50.0)	0	0	50 (28.1)
AK	6 (18.75)	6 (8.8)	12 (28.6)	4 (20.0)	0	0	0	0	28 (15.7)
TOB	6 (18.75)	8 (11.8)	8 (19.0)	6 (30.0)	6 (75.0)	1 (25.0)	0	0	35 (19.7)
PB	0	0	0	0	0	0	0	0	0

**Table 3:** Distribution of enzyme producing isolates.

Specimen types (total number of isolates)	ESBLs Number (%)	MBLs Number (%)	AmpC Number (%)
Pus (32)	13 (40.6)	6 (18.7)	4 (12.5)
Sputum (68)	22 (32.4)	22 (32.3)	4 (5.9)
Urine (42)	12 (28.6)	14 (33.3)	8 (19.0)
Blood (20)	6 (30.0)	6 (30.0)	2 (10.0)
Endotracheal tube (8)	2 (25.0)	6 (75.0)	8 (100)
Swab (4)	2 (50.0)	1 (25.0)	2 (50.0)
Bronchoalveolar lavage (2)	2 (100)	0	0
Pleural fluid (2)	0	0	0

*Total (178)*	*59 (33.1*%)	*55 (30.9*%)	*28 (15.7*%)

**Table 4:** Multitype enzymes production.

Specimen types (total number of isolates)	ESBL + MBL (Number)	ESBL + AmpC (Number)	MBL + AmpC (Number)	ESBL + MBL + AmpC (Number)
Pus (32)	0	0	4	0
Sputum (68)	2	2	2	0
Urine (42)	0	2	6	0
Blood (20)	2	0	0	0
Endotracheal tube (8)	2	2	2	0
Swab (4)	0	0	0	2
Bronchoalveolar lavage (2)	0	0	0	0
Pleural fluid (2)	0	0	0	0

*Total (178)*	*6 (3.4*%)	*6 (3.4*%)	*14 (7.9*%)	*2 (1.1*%)

## Abbreviations

CLSI: Clinical and Laboratory Standard Institute
ESBLs: Extended spectrum beta-lactamases
MBLs: Metallo-beta-lactamases
AmpC: AmpC beta-lactamase
IPD: Inpatient department
OPD: Outpatient department
ASM: American Society for Microbiology
NCCLS: National Committee for Clinical Laboratory Standards
EDTA: Ethylene diamine tetra-acetic acid
MRSA: Methicillin resistant* Staphylococcus aureus*
VRE: Vancomycin resistant* Enterococcus*
GISA: Glycopeptide intermediate* Staphylococcus aureus*
GRSA: Glycopeptide resistant* Staphylococcus aureus*
CAZ: Ceftazidime
CTR: Ceftriaxone
CTX: Cefotaxime
CFM: Cefixime
IPM: Imipenem
MRP: Meropenem
CB: Carbenicillin
PI: Piperacillin
PIT: Piperacillin/tazobactam
CIP: Ciprofloxacin
GEN: Gentamicin
AK: Amikacin
TOB: Tobramycin
PB: Polymyxin B.
